# Radiosurgery for unruptured brain arteriovenous malformations in the pre-ARUBA era: long-term obliteration rate, risk of hemorrhage and functional outcomes

**DOI:** 10.1038/s41598-020-78547-0

**Published:** 2020-12-08

**Authors:** Iulia Peciu-Florianu, Henri-Arthur Leroy, Elodie Drumez, Chloé Dumot, Rabih Aboukaïs, Gustavo Touzet, Xavier Leclerc, Serge Blond, Jean-Paul Lejeune, Nicolas Reyns

**Affiliations:** 1 Department of Neurosurgery, Univ.Lille, CHU Lille, 59000 Lille, France; 2 ULR 2694 - METRICS: Évaluation des Technologies de Santé et des Pratiques Médicales, Univ. Lille, CHU Lille, 59000 Lille, France; 3 Department of Neurosurgery, CHU Lyon, 69000 Lyon, France; 4 Department of Neuroradiology, Univ. Lille, CHU Lille, 59000 Lille, France; 5 Department of Neurosurgery, Lille University Hospital, Rue Emile Laine, 59037 Lille Cedex, France

**Keywords:** Neurology, Prognosis, Therapeutics

## Abstract

The management of non-hemorrhagic arteriovenous malformations (AVMs) remains a subject of debate, even more since the ARUBA trial. Here, we report the obliteration rate, the risk of hemorrhage and the functional outcomes after Gamma Knife radiosurgery (GKRS) as first-line treatment for non-hemorrhagic AVMs treated before the ARUBA publication, in a reference university center with multimodal AVM treatments available. We retrospectively analyzed data from a continuous series of 172 patients harboring unruptured AVMs treated by GKRS as first-line treatment in our Lille University Hospital, France, between April 2004 and December 2013. The primary outcome was obliteration rate. Secondary outcomes were the hemorrhage rate, the modified Rankin Scale (mRS), morbidity and epilepsy control at last follow-up. The minimal follow-up period was of 3 years. Median age at presentation was 40 years (IQR 28; 51). Median follow-up was 8.8 years (IQR 6.8; 11.3). Median target volume was 1.9 cm^3^ (IQR 0.8–3.3 cm^3^), median Spetzler-Martin grade: 2 (IQR 1–2), median Pollock-Flickinger score: 1.07 (IQR 0.82–2.94), median Virginia score: 1 (IQR 1–2). Median treatment dose was 24 Gy at 50% isodose line. Twenty-three patients underwent a second GKRS after a median time of 58 months after first GKRS. The overall obliteration rate was of 76%, based primarily on cerebral angiography and/or rarely only upon MRI. Hemorrhage during the post-treatment follow-up was reported in 18 (10%) patients (annual risk of 1.1%). Transient post-GKRS morbidity was reported in 14 cases (8%) and persistent neurological deficit in 8 (4.6%) of patients. At last follow-up, 86% of patients had a mRS ≤ 1. Concerning patients with pretherapeutic epilepsy, 84.6% of them were seizure-free at last follow-up. GKRS as first-line therapeutic option for unruptured cerebral AVMs achieves high obliteration rates (76%) while maintaining a high-level patient’s autonomy. All hemorrhagic events occurred during the first 4 years after the initial GKRS. In cases with epilepsy, there was 84.6% seizure free at last follow-up. Permanent morbidity was reported in only 4.6%.

## Introduction

Cerebral arteriovenous malformations (AVMs) represent a rare pathology in the general population^1^, with an incidence of approximately 1:100,000^2^. These malformations can be associated with a noteworthy morbidity, even mortality due to the potential occurrence of massive intracranial bleeding^3,4^. The risk of bleeding is variable, between 0.9% to as high as 35%^4^, depending on several factors described as independent predictors for hemorrhage: increasing age, initial hemorrhagic AVM presentation, deep brain location, and exclusive deep venous drainage^5,6^. In the case of a hemorrhagic event consensus can be easily reached to promote the earliest possible treatment of the vascular nidus, thus reducing the subsequent risk of new bleeding. Classical treatment options might include microsurgical resection^7^, endovascular approach^8,9^ or stereotactic radiosurgery^10–12^. These treatment modalities can be performed stand-alone or combined, depending on the AVM architecture and the post-therapeutic evolution.

In the particular case of an unruptured AVM with no previous history of bleeding, the optimal patient management remains a matter of debate. The publication of the ARUBA trial represented a turning point, generating a paradigm shift in the multidisciplinary management of such particular cases^13^. Mohr et al. reported that medical management alone is superior to medical management with interventional therapy, including surgery / embolization / radiosurgery, for the prevention of death or stroke in patients with unruptured brain arteriovenous malformations^13^. However, the conclusions of this study are questioned by limiting factors such as a short follow-up and a disproportionate number of patients treated with surgery and embolization. In more recent studies, some authors advocate for an interventional treatment of unruptured AVM, arguing their results with radiosurgery or microsurgery are superior to conservative management in terms of reducing the risk of stroke and death^14,15^. Balancing the risk of hemorrhage, a European consensus promoted the treatment of unruptured brain AVM Spetzler-Martin grades 1 and 2^16^, in a multidisciplinary and multimodal approach^17^. Identifying the optimal patient profile for treatment even in the case of an unruptured AVM remains so a major challenge.

The primary objective of the current study was to report the obliteration rate of unruptured AVMs treated by Leksell Gamma Knife radiosurgery (GKRS) in our university center before the ARUBA trial publication. The secondary objectives were the hemorrhage rate, the modified Rankin Scale (mRS), morbidity and epilepsy control at last follow-up.

## Results

### Basic demographic data

The median age was 40 years (IQR 28; 51). The male/female ratio was of 0.85. Most patients presented with epilepsy [80 (46%)] or headaches [42 (24%)]. Thirty-six (21%) patients were incidentally diagnosed. A comprehensive list of initial symptoms can be found in Table 1.

**Table 1:** Basic demographic data.

Variables	Values
**Population**	
Age (years), (median, IQR)	40 (28; 51)
**Sex**	
Male	79 (46)
Female	93 (54)
M/F ratio	0.85
**Initial symptom(s)**	
Epilepsy	80 (46)
Headaches	42 (24)
Incidental	36 (21)
Neurological deficit	10 (6)
Vertigo, dizziness	4 (2)
Paresthesia	3 (2)
Follow-up (year) (median, IQR)	8.8 (6.8; 11.3)
**AVM characteristics**	
Location	
Lobar	
Frontal	55 (32)
Insular	4 (2)
Temporal	44 (26)
Parietal	18 (10)
Occipital	23 (14)
Posterior fossa	8 (5)
Deap seated	20 (12)
Eloquent area	111 (65)
Pollock Flickinger score (median, IQR)	1.087 (0.82; 1.31)
Virginia score (median, IQR)	1 (1; 2)
0	33 (19)
1	70 (41)
2	47 (27)
3	22 (13)
Spetzler-Martin grade (median, IQR)	2 (1; 2)
1	48 (28)
2	84 (49)
3	38 (22)
4	2 (1)
5	0 (0)

Values are presented as frequency (percentage) unless otherwise indicated.

The treated AVM were mainly lobar [frontal: 55 (32%), temporal: 44 (26%)]. Twenty (12%) were deep-seated (basal ganglion, thalamus, mesencephalon). Eloquent areas were involved in 111 (65%) cases. AVM drainage was superficial in 135 (78%), deep in 25 (15%) and mixt in 12 (7%) patients. Thirteen (8%) AVM harbored an intra or perinidal aneurysm. The median Pollock-Flickinger Score was 1.087 (IQR 0.82; 1.31). The median Virginia Score was 1 (IQR 1; 2). The median Spetzler-Martin Grade was 2 (IQR 1; 2) (Table 1).

In the case of a persistent circulating AVM, 23 (13%) patients underwent a second GKRS treatment, after a median time of 58 months (IQR 46; 66.5).

The median follow-up was 8.8 years (IQR 6.8; 11.3).

### Basic dosimetric data

#### First GKRS

The median target volume (TV) was 1.9 cm^3^ (IQR 0.8–3.3) with a prescribed marginal dose of 24 Gy (IQR 18–27) at the 50% isodose line. The median V12 was 4.5 cm^3^ (IQR 2.2–8.9) and the gradient index was 2.77 (IQR, 2.56–3.03).

#### Second GKRS

The median TV was 0.4 cm3 (IQR 0.21–1), with a prescribed marginal dose of 24 Gy (IQR 21–24) at the 50% isodose line. The median V12 was 1.44 cm^3^ (IQR 0.8–4) and the gradient index was 2.8 (IQR 2.62–3.18) (Table 2).

**Table 2:** Basic dosimetric data (med = median, IQR = interquartile range).

Variables	1st GKRS (n = 172)	2nd GKRS (n = 23)
	Median (IQR)	Median (IQR)
Target volume in cm^3^ (med, IQR)	1.9 (0.8; 3.3)	0.4 (0.21; 1)
Marginal dose (med, range)	24 (18; 27)	24 (21; 24)
V12 (med, IQR)	4.5 (2.2; 8.9)	1.44 (0.8; 4)
Gradient index (med, IQR)	2.77 (2.56; 3.03)	2.8 (2.62; 3.18)

### Obliteration rate

**Figure 1:**
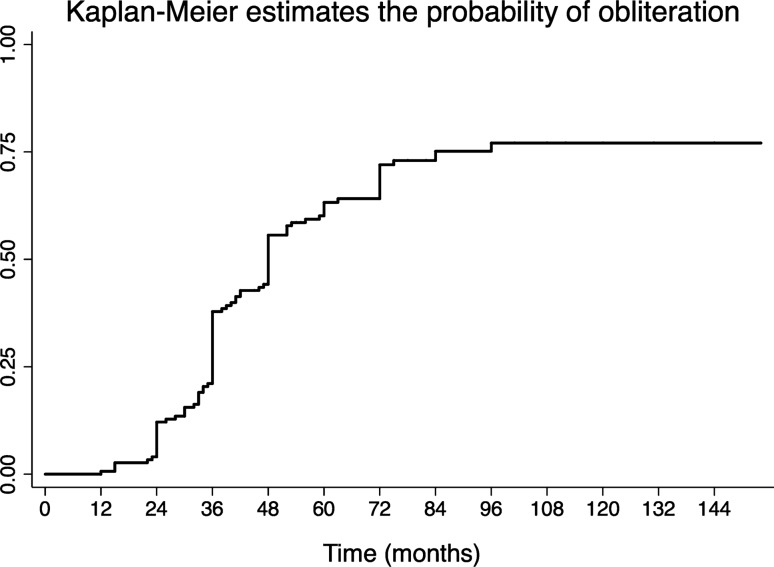
Actuarial obliteration rates, with n at risk being 151 at 2 years, 84 at 4 years, 52 at 6 years, 31 at 8 years, 14 at 10 years, 5 at 12 years and 0 at 14 years.

The final obliteration rate after the first GKRS was 76% after a follow-up of 12 years (Fig. 1). The median time to occurrence was 4.3 years (IQR 3–12).

Among the 23 patients who received a second GKRS treatment on the residual nidus, the final obliteration rate was 100% after a follow-up of 10 years.

No association was reported between the probability of obliteration and the following variables: age, sex, Pollock-Flickinger Score, Virginia Score, Spetzler-Martin Grade, Target Volume and the V12 (Table 3).

**Table 3:** Associated factors with the different endpoints.

Variables	HR (95% CI)	*p* value
**Obliteration rate (112 patients, 76%)**		
Age	1.01 (0.99–1.02)	0.2
Sex	0.76 (0.5–1.1)	0.18
Pollock–Flickinger score	0.88 (0.52–1.5)	0.66
Virginia score	0.94 (0.76–1.16)	0.5
Spetzler-Martin grade	0.88 (0.67–1.16)	0.37
Target volume	0.96 (0.87–1,05)	0.36
V12	0.99 (0.96–1.03)	0.7
**Hemorrhage rate (18 patients, 10%)**		
Pollock–Flickinger score	**3.34 (1.39**–**8.05)**	**0.007**
Virginia score	1.59 (0.97–2.61)	0.06
Spetzler–Martin grade	1.29 (0.69–2.39)	0.42
Target volume	**1.23 (1.07**–**1.43)**	**0.005**
V12	**1.08 (1.02**–**1.13)**	**0.006**

mRS	Correlation coefficient	*p* value
Eloquent area	/	0.97
Pollock–Flickinger score	**0.15**	**0.050**
Virginia score	0.15	0.053
Spetzler–Martin grade	0.04	0.59
Target volume	**0.17**	**0.037**
V12	**0.20**	**0.020**

Bold values correspond to significant statistical differences.

### Hemorrhage rate

**Figure 2:**
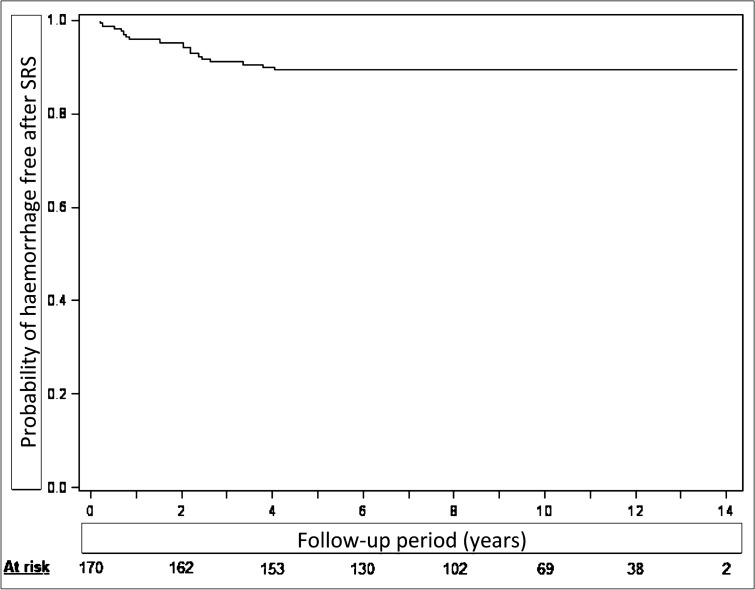
Actuarial probability of hemorrhage free after GKRS, with n at risk being 162 at 2 years, 153 at 4 years, 130 at 6 years, 102 at 8 years, 69 at 10 years, 38 at 12 years and 2 at 14 years. The area under the curve represent the absence of AVM bleeding during the follow-up”.

Eighteen (10%) patients harbored intracranial hemorrhages during the follow-up (Fig. 2). All bleedings occurred during the follow-up after 1st GKRS. Fifteen hemorrhagic events happened in the first 3 years after GKRS. Only 3 patients bled after 3 years of follow-up (respectively at 3.3 years, 3.75 years and 4 years). The annual risk of bleeding is 1.1% (Fig. 2). When stratified before/after 4 years of follow-up, the annual risk of bleeding is 2.6% before 4 years, and 0% after 4 years. Four (2%) patients died due to the acute bleeding. All intracranial bleedings occurred during the 48 months after initial GKRS. In our series, no hemorrhage occurred after documented nidus obliteration.

The Pollock-Flickinger Score was associated with the hemorrhage risk (HR = 3.34, 95% CI 1.39–8.05, p = 0.007) (Table 3). For each 0.5-point increase of Pollock-Flickinger Score, the risk of bleeding raised by 1.8 (95%CI, 1.18–2.84). The TV was also associated with the bleeding risk (HR for each additional cm^3^ = 1.23, 95%CI 1.07–1.43, p = 0.005). In the same manner, an increase of the V12 is associated with an increase of the hemorrhage rate, (HR = 1.08, 95%CI 1.02–1.13, p = 0.006). A tendency was reported regarding The Virginia score (p = 0.06). No statistical association was reported with the Spetzler–Martin Grade (p = 0.42).

### Clinical outcomes

Epilepsy, the main initial symptom, was cured in 84.6% of patients after GKRS, which were seizure free at last follow-up.

Transient post-GKRS morbidity was reported in 14 cases (8%) (Table 4). These transient symptoms were mostly associated with perilesional edema on the MRI, reducing spontaneously or with corticosteroids. In patient without bleeding during follow-up, persistent neurological deficits were reported in 8 (4.6%) patients (Table 4). As previously mentioned, 4 (2%) patients died due to the acute bleeding during the follow-up after GKRS treatment. Two other patients presented with permanent motor deficit after bleeding. The modified Rankin Scale (mRS) was ≤ 1 in 86% of patients at last follow-up. Correlations between a worsening of the mRS and the following variables were reported in Table 3: increase of Pollock Flickinger score (p = 0.050), increase of Virginia Score (p = 0.053), higher TV (p = 0.037) and higher V12 (p = 0.020). No correlation was found with AVM location in eloquent area (p = 0.97) or Spetzler Martin grade (p = 0.59).

**Table 4:** Transient post-GKRS morbidity and persistent neurological deficits.

Variables	Values
Transient morbidity—total	**14 (8)**
Cephalalgia	6 (3.5)
Epilepsy—seizure exacerbation	5 (2.9)
Intermittent sensory deficit	1 (0.6)
Speech disturbance	1 (0.6)
Vertigo	1 (0.6)
Permanent deficit—total	**8 (4.6)**
Homonymous lateral hemianopia	2 (1.2)
Motor deficit (hemiparesis or segmental deficit)	1 (0.6)
Hypoesthesia (segmental deficit)	1 (0.6)
Cognitive decline	2 (1.1)
Superior lateral quadranopia	1 (0.6)
Ataxia	1 (0.6)

Bold values correspond to significant statistical differences.

Values are presented as frequency (percentage) unless otherwise indicated.

## Discussion

Here, using inclusions criteria for brain AVM such as those reported by the ARUBA trial, we report an initial obliteration rate as high as 76%. After including a second GKRS, the obliteration rate was of 100%. Hemorrhage during the post-treatment follow-up was reported in 18 (10%) patients. All hemorrhagic events occurred in the first 4 years after initial GKRS. Transient post-GKRS morbidity was reported in 14 cases (8%) and persistent neurological deficit in 13 (7.5%) of patients. At last follow-up, 86% of patients had a mRS ≤ 1 and 88% of them were free of symptoms. Epilepsy, the main symptom at discovery, disappeared in more than 80% of the patients, who were seizure free at last follow-up.

The ARUBA trial was the first multicenter randomized trial on the treatment benefit for non-hemorrhagic AVMs, published in 2014, analyzing a total of 223 patients^13^. In their conclusions the authors stated that the risk of death or stroke was significantly lower in the medical management group than in the interventional therapy group (hazard ratio 0.27, 95% CI 0.14–0.54). Many questions arose after the publication of these results. One of the main debates concerned the pooled analysis of all interventional procedures, mixing a variety of treatments: surgical excision, endovascular treatment and radiosurgery with different respective morbidity^18^. Moreover, in the ARUBA study, the follow-up was considered too limited (mean of 33.3 months) to fully assess the benefits of each treatment and even more so of radiosurgery, which reaches maximum efficiency in several years^18^.

The ARUBA findings were supported by the publication of the Scottish Audit of Intracranial Vascular Malformations study that compared the long-term outcomes between conservative management and interventional treatment for unruptured cerebral AVMs^19^. This non-randomized study postulated that adult patients could benefit more from conservative treatment that interventional (all types of treatment included, single or multimodal) but only 27% of patients on the interventional arm were treated only by radiosurgery, with a reported 64% complete obliteration (as stated in Table 1 from online Supplement from the same manuscript).

Several series have also previously reported their results in terms of obliteration rates and safety-efficacy profiles in patients with unruptured AVM^20^. Tonetti et al.^20^ suggested that after a sensible follow-up period exceeding the latency period, there is a lower rate of stroke/death for patients with treated, unruptured AVMs with SRS as compared to those left untreated. Ding et al. suggested up to 87% of obliteration at 10 years and 1% of permanent radiation-induced changes, proposing active treatment for low-grade AVMs in patients with a life expectancy of minimum 10 years^21^. Furthermore, in their publications, both Starke et al. and Ding et al. reported smaller volumes and non-eloquent location as independent prognosis factor for complete obliteration^22,23^. In our series, in the case of small nidus (Spetzler Martin 1or 2) located in non-eloquent area, when it was conceivable, surgery and radiosurgery were proposed as alternative treatments to the patient. According to our retrospective data, 24 (14%) AVM harbored a Pollock Flickinger ≥ 1.5, 22 (13%) AVM presented with a Virginia Score ≥ 3. Among these patients, 21 had a SM grade, ≤ 3. In these 21 patients, surgery could have been an alternative as first-line treatment. Using GKRS, in this subgroup, the obliteration rate was 68%. These data emphasized that almost 90% of the unruptured AVM in this study harbored favorable Pollock-Flickinger score and Virginia score to be treated efficiently with GKRS. Low SM grade (in particular due to the small nidus size) is also a good criterion for GKRS efficacy, mainly for deep location. Even though in our study none of these factors reached this level of statistical significance, future subgroup analysis and eventually a larger population are needed. In their study, Ding et al. showed that good obliteration can be achieved in highly eloquent areas where the ideal microsurgical excision can result in high morbidity^24^. The authors further estimated that a follow-up duration of 15 to 20 years is mandatory to realize a potential benefit of radiosurgical intervention for conservative management in unruptured patients with AVM. In our series, although 65% of AVM were located in eloquent areas, 86% of patients had a mRS ≤ 1 after 8.8 years of follow-up. This result reflects the low morbidity of GKRS as a treatment option for unruptured AVM.

In our experience, we favor a targeted based on stereotactic DSA for each treated case.

We tend to limit the targeted volume to a clearly visualized arteriovenous shunt. As previous studies have shown a relationship between adverse radiation effects and the V12 volume, we further evaluate the V12 also. If the AVM was not obliterated after a first GKRS, with a minimal follow-up of 3 years and depending on the imaging evolution of the nidus, we perform a second one on the residual shunt. This type of approach, as reflected in the present paper, proved to have limited toxicity. Thus, we favor a confined volume for the first GKRS and, if further needed, we perform the second.

We did not identify any associated factor with obliteration (Table 3). A contrario, the 24% of unobliterated AVM at the end of the follow-up did not differ from the rest of the study population. As during the study period, the same practitioners (SB, NR, GT) performed the GKRS treatments, the criteria of patients’ selection, targeting, and dosimetry were homogeneous (Tables 1, 2). That could explain the absence of such statistical difference in our series. In our cohort, the median dose was of 24 Gy (IQR 24; 24). The prescription dose was very homogenous in the study and as a consequence could not be analyzed as a variable of bleeding or obliteration. However, we recently reported that the biologically effective dose (BED) is more predictive of GKRS efficiency than the prescribed dose itself^25^.

We report an annual hemorrhage rate of 1.1%. Ding et al.^15^ recently suggested in a multicentric international retrospective cohort study that the annual post SRS hemorrhage rate was 1.4%, which is fairly comparable to our present research. Tonetti et al.^20^ reported an annual hemorrhage rate after 3 years of follow-up of 0.4% (26 occurrences in a series of 233, 16 patients died as a result, at last follow-up). In a recent published cohort of 264 AVM, Kim et al. reported an annual hemorrhage rate of 3.4%. This higher percentage of hemorrhage could be explained by the lower dose (median 20.8 Gy) distributed to bigger nidus (median 4.8 cm^3^)^26^. In our series, the 18 hemorrhagic events (2% mortality) occurred during the first 4 years after the initial GKRS. It emphasized the importance of close clinical and imaging monitoring until full AVM occlusion.

Similarly to Ding et al., we reported transient or permanent symptomatic radiation-induced changes in 9% and 3%, respectively^15^.

With regards to epilepsy control, we reported seizure free rates of approximately 80%, while this being the most common symptom in our study’s population. In their study, Ding et al. found that lack of previous AVM hemorrhage (p < 0.0001), larger nidus diameter (p < 0.0001), and cortical AVM location (p < 0.0001) were independent predictors of seizure presentation^27^. Chen et al. proposed as strong predictors for pre-treatment seizures a previous AVM resection, a cortical AVM location, and a lack of previous AVM hemorrhage^28^. Przybylowski et al. showed in their retrospective study that effective long-term results can be seen, with 89% remission (Engel class IA or IB), especially in the case of simple partial or secondarily generalized seizures^29^. In their meta-analysis, Baranoski et al. demonstrated that radiosurgery can be very effective in good seizure outcome, even more so if complete obliteration is achieved (62.8% and 85.2% respectively)^30^. Seizure-free rates after SRS for AVMs in eloquent areas have been reported as early as the nineties’^31^.

### Study limitations

The main limitations of our study were its retrospective nature. As many patients were referred from different neurosurgical centers their follow up was difficult to obtain. Therefore, some delays of obliteration may be overestimated. Larger population databases with strict follow up protocols or multicentric studies are needed to increase the weight of statistical analysis and confirm our favorable results. Other limitation is that part of the patients underwent brain MRI to conclude to obliteration. In this respect, MRI is not considered as being as such sensitive and specific as the DSA and could overestimate the rate of obliteration.

## Conclusion

The optimal treatment of unruptured AVMs remains debatable but newer studies confirm the beneficial role of treating unruptured AVMs by SRS, as part of a multidisciplinary algorithm and a tailor management, adapted to individual patient’s profile.

With an overall obliteration rate of 76%, a permanent morbidity rate of 4.6% and an annual bleeding risk at 1.1%, our results confirm the beneficial role of radiosurgery for unruptured AVMs, with a fair profile of safety and efficacy. In our series, a major finding was that no hemorrhage occurred after the first 4 years following initial GKRS.

## Methods

All methods were carried out in accordance with the applicable guidelines (STROBE statement), in compliance with the Declaration of Helsinki. Our institutional ethical committee approved this study (Comité d’éthique pour la recherche de l’Université de Lille, ID:783). The written informed consent was obtained from all the patients (or their legal representant) in the study.

### Patient population

We retrospectively included a continuous series of 172 patients harboring unruptured cerebral AVM treated in our university hospital from the beginning of our radiosurgical activity using Leksell Gamma Knife model 4C (Elekta Instruments, AB, Sweden) from April 2004 to December 2013.

The inclusion criteria matched the inclusion criteria of the ARUBA trial adapted to the current trial design: age > 18 years old, patients diagnosed with non-hemorrhagic brain AVM (i.e. no clinical or radiological signs of prior hemorrhagic events), solely treated by GKRS as first-line option and second-line option (in case of the persistence of AVM nidus more than 3 years after the 1st GKRS), all AVM locations, with minimal follow-up of 3 years. In our university center, the treatment modality choice for unruptured AVM relied on a multidisciplinary approach, including neurosurgeons, neuroradiologists and neurologists. Before performing GKRS, every case was reviewed by an expert radiosurgeon (SB, NR).

The exclusion criteria were: prior hemorrhage (clinical or radiological patterns), prior treatment attempt (i.e. surgery or embolization), pediatric patients, volume staged GKRS, other associated vascular abnormality (cavernous malformation, dural arteriovenous fistula, multiple foci AVM), coagulopathy, thrombocytopenia.

The basic demographic data are presented in Table 1.

### Radiosurgical technique

All patients underwent GKRS with Leksell Gamma Knife 4C model (Elekta Instruments, AB, Stockholm, Sweden). The Leksell stereotactic frame was applied under local anesthesia, followed by a stereotactic MRI, CT scan and digital subtraction cerebral angiography, all performed the same day.

All patients benefitted from a 3D T1 gadolinium enhanced sequence, in thin slices (≤ 1 mm) and T2.

Digital subtraction cerebral angiography (DSA) was further performed with a 3 to 6 acquisition per-second speed depending on the blood velocity inside the nidus, also under local anesthesia, through a femoral approach, to define the dynamic anatomy and spatial angioarchitecture of the nidus for each patient. This supplementary exam involved different vascular axes injection, depending on arterial feeder for each cerebral AVM (internal carotid artery, vertebral artery). The sequences were then transferred to the Gamma Plan in a 2D recording.

Our philosophy is similar to the Pittsburgh team, as we do not routinely contour AVMs on structure MR imaging. We first define the nidus to target on the 2D DSA images and then compare its location on the 3D MRI images.

In case of persistent AVM nidus visualized on DSA at least 3 years after initial treatment, a second GKRS was performed in 23 patients.

### Follow up

Standard follow-up protocol in our center always involves clinical and radiological assessment, performed regularly in a 6–12 months interval for the first 2 years and in a yearly manner afterwards. For each patient, the minimum follow-up was of three years.

Radiological follow-up was initially performed by cerebral MRI with usual 3D T1 gadolinium contrast enhanced and T2 sequences before each clinical consultation. Supplementary neuroimaging was performed between scheduled controls if neurologic symptoms developed after GKRS. Whenever possible complete obliteration was confirmed by a cerebral DSA, as the gold standard for this primary outcome, performed more than three years after initial GKRS.

### Statistical analysis

Qualitative variables are expressed as numbers (percentage). Quantitative variables are expressed as the median (interquartile range (IQR)). Normality of distributions was assessed using histograms and Shapiro–Wilk test. The obliteration and hemorrhage rates after GKRS were estimated using the Kaplan–Meier method. Associations of several factors with obliteration and hemorrhage rates after GKRS were evaluated using Cox proportional hazard regression models. Hazard ratio (HR) and its 95% confidence interval (CI) were reported as effect size measure. For mRS, associations with several factors were evaluated using the Spearman correlation coefficient for quantitative factors and using a Mann–Whitney *U* test for eloquent area. Statistical testing was done at the two-tailed α level of 0.05. Data were analyzed using the SAS software package, release 9.4 (SAS Institute, Cary, NC).

### Ethical approval

All procedures performed in studies involving human participants were in accordance with the ethical standards of the institutional and/or national research committee (name of institute/committee) and with the 1964 Helsinki declaration and its later amendments or comparable ethical standards.
